# Molecular characterization of chromium tolerant and gelatin hydrolyzing bacterial isolates from tannery wastes: Perspective on chrome-tanned leather waste biodegradation in Bangladesh

**DOI:** 10.1016/j.jgeb.2025.100479

**Published:** 2025-03-28

**Authors:** Shashanka Shekhar Sarker, Md.Murshed Hasan Sarkar, Shamima Akhter Sharmin, Nourin Tarannum, Taslima Akter, Md.Ashraful Alam, Md.Ibrahim Miah, Md.Aftab Ali Shaikh, Sahana Parveen

**Affiliations:** a Leather Research Institute (LRI), Bangladesh Council of Scientific and Industrial Research (BCSIR), Nayarhat, Savar, Dhaka 1350, Bangladesh; b Bangladesh Council of Scientific and Industrial Research Laboratories, Dhaka 1205, Bangladesh; c Environmental Biotechnology Division, National Institute of Biotechnology (NIB), Ganakbari, Ashulia, Savar, Dhaka 1349, Bangladesh; d Department of Microbiology, University of Dhaka, Dhaka 1000, Bangladesh; e Bangladesh Council of Scientific and Industrial Research (BCSIR), Dhanmondi, Dhaka 1205, Bangladesh; f Department of Chemistry, University of Dhaka, Dhaka 1000, Bangladesh

**Keywords:** Tannery waste, Bacteria, Cr tolerance, Gelatin-hydrolysis, CTLW biodegradation

## Abstract

Improper management of chrome-tanned leather waste (CTLW) might potentially cause adverse environmental consequences. To mitigate that harmful impact, this study aims to find and conduct molecular characterization of bacteria from tannery wastes that can tolerate chromium (Cr) and hydrolyze gelatin. Bacteria from tannery wastes are naturally adapted to Cr; eight Cr(III) tolerant bacteria, namely bacterial isolate (BI) 1 to 8, were isolated from the collected waste samples. The isolated bacteria showed the maximum tolerance concentration (MTC) range of 700 to 1500 ppm for Cr(III) and 200 to 600 ppm for Cr(VI). Physiological and biochemical analysis, including the gelatin hydrolysis activity, identified those isolates up to the genus level. Among the isolates, BI 4, 5, and 7 were able to hydrolyze gelatin. Therefore, 16S rRNA molecular characterization was conducted for those isolates, which confirmed BI 4, 5, and 7 as *Bacillus wiedmannii* (Accession No: OR564007), *Enterococcus faecium* (Accession No: OR564008), and *Bacillus cereus* (Accession No: OR564009), respectively. Bacteria with gelatin hydrolyzing activity can be the potential for degrading hydrothermally treated CTLW. Thereby, those three isolates were applied to explore their biodegradation ability in real world scenario. The biodegradation experiments showed that *Enterococcus faecium*, *Bacillus cereus*, and *Bacillus wiedmannii* were able to biodegrade hydrothermally treated CTLW at 98.67 %, 98.33 % and 98.00 %, respectively. The present study demonstrates *Enterococcus faecium*, *Bacillus cereus*, and *Bacillus wiedmannii* having biodegradation of CTLW applications might mitigate environmental pollution caused by this waste in the perspective of Bangladesh.

## Highlights

• CTLW is responsible for environmental pollution.
• Bacteria isolated from tannery wastes are resistant to Cr.
• Cr-tolerance determination helps find bacteria potential for waste management.
• Bacteria with gelatin hydrolyzing ability can be applied for CTLW degradation

## Graphical abstract

**Figure:**
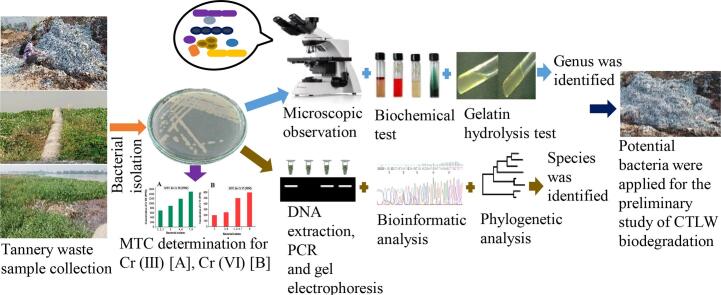

## 1 Introduction

Leather holds a prominent position in Bangladesh's national economy and has a good reputation around the world. The leather industry and its producers are essential and reliable contributors to export trade and a significant source of foreign exchange revenues [1]. The export of leather from Bangladesh experienced a significant growth of 30.95 % during the period from July 2021 to March 2022, in comparison to the corresponding period in the previous fiscal year. This resulted in a total revenue of $896.8 million, equivalent to BDT 9,510 crore. By 2030, the government wants to increase the export earnings from the leather industry from less than $1 billion to $10–12 billion. To that end, it creates a ten-year perspective plan [2]. To meet that target, about 113 tanneries are actively functioning [3]. However, the increasing worry over environmental pollution caused by tannery waste is considered one of the most crucial limitations for establishing tannery industries [1]. A lot of different chemicals are used at various stages of leather production to turn raw skins and hides into commercially valuable leather [4]. Chromium sulfate salts, a widely used chemical and a significant tanning agent, are used to increase the stability and durability of leather [5]. During chrome tanning, Cr(III) ions form coordinated cross-links with carboxyl groups of aspartic and glutamic acids in collagen render skins/hides highly stable [6]. Around 60–70 % of applied Cr salts undergo a reaction with the hide and skin, while the remaining portion is retained in the form of solid and liquid waste [7]. Cr is present in the solid debris generated by shaving, buffing, trimmings and splits leather, together called leather dust, produced when chrome-tanned crust leather is converted into polished leather. This chrome-tanned leather waste (CTLW) accounts for about 35–40 % of solid wastes, typically containing 3 % Cr2O3, 90 % collagen, and 7 % other impurities 4, 8, 9. The available methods for CTLW management include landfill and incineration. These conventional methods are expensive and adversely affect the environment due to the leaching of Cr ions, oxidation of Cr(III) into Cr(VI) during thermal incineration, and production of noxious emits, specifically nitric oxide [10]. Besides, CTLW is used as the principal component of poultry feed, and fish feed because they are rich in protein content [11]. Thus, Cr enters the food chain and is transported from tannery wastes to the human body, which causes toxic effects on human beings, including brain damage, lung disease, liver fibrosis, kidney damage, neurotoxic effects, and even cancer 7, 12. Cr(III), the main component of basic chromium sulfate is less hazardous than Cr(VI) [13]. Nevertheless, inside the living being Cr(III) species can be metabolized into Cr(VI). Thus, Cr(III) become potentially hazardous for the health [14]. European Food Safety Authority (EFSA) did not recommend the daily consumption of Cr(III) due to inadequate evidence of its health advantages. However, a permissible weekly dosage of 300 μg/kg of body weight has been established [13]. Extended exposure to Cr(III) can lead to the development of skin allergies and cancer [15]. To solve these hazardous issues and boost tannery output, an environmentally friendly approach of CTLW management is urgently needed. Its management through microbial means can be an eco-friendly approach and alternative to the conventional methods. The potential use of microorganisms in solid waste treatment is now being investigated, aiming to mitigate or eliminate pollutants and facilitate the recycling or reutilization of waste materials [16]. To lessen the toxicity of CTLW, the Cr bound within the collagen matrix must be removed, and this can be achieved by breaking the bond between Cr and collagen through bacterial degradation of leather by protease activity such as gelatinase 17, 18. Pretreatment like autoclaving can cause thermal denaturation of the collagen structure, which might result in the formation of gelatin. Gelatinase producing bacteria can utilize the produced gelatin as a substrate for their efficient growth, and can be successful at degrading fibrous proteins including collagen, causing the degradation of CTLW, and liberating Cr in the liquefied effluent 4, 18; Cr can then be recovered from the effluent [19]. In order to the bacterial growth of chrome-tanned leather waste and its degradation, the strain must have the ability to grow in an environment that contains high concentrations of Cr[20]. A number of microorganisms have evolved heavy metal resistance. Various types of bacteria, including *Bacillus subtilis* 4, 18, *Alcaligenes faecalis* [17], *Lactobacillus* strains [21], *Enterococcus faecium* [22], *Bacillus amyloliquefaciens* 23, 24, *Bacillus cereus* [25], *Bacillus methylotrophicus* [26], *Bacillus proteolyticus* [27], *Staphylococcus sciuri* [28], *Acinetobacter* sp.[29], *Arthrobacter* sp.[30], *Pseudomonas* sp.[31], *Cellulomonas* sp. [32], *Escherichia coli* [33], *Enterobacter cloacae* [34], *Staphylococcus aureus* and some species of *Klebsiella* [35] have been found to show resistance towards Cr. These bacteria have the capacity to protect themselves against the harmful effects of heavy metals through several mechanisms, such as adsorption, absorption, methylation, oxidation, and reduction [36]. Besides, chromium resistant bacteria exhibited a wide range of mechanisms to cope with the stress induced by chromium for their survival. These mechanisms are exopolysaccharide (EPS) secretion, Cr(III) adsorption of lipopolysaccharide (LPS), bioaccumulation, efflux system, and Reactive Oxygen Species (ROS) detoxification [37]. The ability of the microbes to withstand heavy metals like Cr make their isolation, Cr-tolerance characterization, exploring gelatin hydrolysis ability, and identification important. Thus, the current research focuses on the isolation of bacteria from tannery wastes, their characterization for Cr tolerance ability, determination of gelatin hydrolysis activity, and identification of potential bacterial isolates. Finally, setting up a small scale biodegradation experiments utilizing bacterial isolates having gelatin hydrolysis activity also considered to explore their ability to biodegrade CTLW. Based on the availability of literature currently in circulation, this research article is the first one ever on the degradation of CTLW utilizing bacteria in Bangladesh. Future schemes will include the establishment of an optimal CTLW biodegradation approach utilizing the potential bacterial species, consequently, recovery of Cr from the liquefied effluent to mitigate environmental pollution caused by CTLW.

## 2 Materials and methods

### 2.1 Study area and sample collection

In this study, the sampling sites were in the Tannery Industrial Estate at Savar, Dhaka, Bangladesh. Chrome-tanned leather waste was collected from the chrome shavings landfill area, drain water (DW) was also collected from the drainage system of the same area, and effluent containing water (ECW) was collected from the surrounding regions of different effluent discharge points. Sterile plastic containers with a capacity of 500 mL and zipper bags were utilized for the collection of liquid and CTLW samples, respectively. The liquid samples were transported on ice to the Microbiology laboratory of Leather Research Institute (LRI) and stored at 4 °C before analysis and during the experiments [38].

### 2.2 Physico-chemical analysis of liquid and solid samples

Physical and chemical analyses of DW and ECW were used to analyze several parameters, including temperature, pH, total dissolved solids (TDS), and total Cr. Temperature and pH were measured employing a multimeter (Loviband SD-50). TDS was measured using a TDS meter (Loviband SD-80). The Cr content of DW, ECW, and CTLW was measured using an inductively coupled plasma optical emission spectrometer (ICP-OES) (Model-5110 ICP-OES, Agilent). The total Cr content was determined by digesting each sample using the microwave digestion procedure USEPA 3015A for DW and ECW 39, 40 and Leather ASTM for CTLW.

In brief, for DW and ECW, 45 mL sample was taken within a 50 mL quartz digestion tube following the addition of 5 mL HNO_3_ (70 %) to the sample. The containers were then sealed and subjected to a two-stage digesting process, which initially heating them for 15 min until a temperature of 170 °C was reached, followed by a second phase lasting 5 min at 170 °C. Each digested product was allowed to cool down and be filtered. The filtrates were analyzed upon ten times dilution with distilled deionized water (DDW).

For CTLW, 0.2 g of fine particle (2 mm in size) sample was taken in a quartz digestion tube with a capacity of 50 and 10 mL of HNO_3_ (70 %), and 1 mL of H_2_O_2_ (30 %) was added to the sample. Following that, the vessels were sealed and heated at three steps. The first step involved heating at 130 °C for 1 min, followed by 170 °C for 1 min at the second step. The third step was lasted for 15 min at 190 °C. Digested products were cooled, filtered, and then diluted to a volume of 100 mL with DDW.

### 2.3 Isolation of Cr(III)-tolerant bacteria: Primary screening

Bacteria from wet CTLW were isolated as previously described method [41] with modifications. A 30 g of dried CTLW was immersed in 50 mL of distilled water in 250 mL beaker and left aseptically for a period of 3 days. Under aseptic conditions, 1 mL of the wet CTLW extract was transferred in 9 mL of sterile 0.85 % saline water, and serial dilution was performed to 10^-2^. A similar serial dilution method was also conducted for DW and ECW samples. A volume of 100 μL from both diluted and undiluted CTLW extract, DW, and ECW samples were aseptically transferred into freshly prepared Nutrient Agar (NA) medium, following the spread plate techniques as previously described 42, 43, 44 with modifications. The medium was amended with 100 ppm of Cr(III) using basic chromium sulfate as a source. The sample was spread aseptically on the surface of the agar medium utilizing the sterile glass spreader. After that, the plates were incubated at 37 °C for 1–4 days. Potential single colonies were selected and sub-cultured by streaking method onto fresh NA medium containing the same concentration of Cr(III) to obtain pure culture, labeled, and kept at 4 °C in a refrigerator and also in 80 % glycerol stock for further investigations.

### 2.4 Phenotypic and biochemical analysis of isolated bacteria

Tentative identification of all bacterial isolates were conducted using colony morphology, growth pattern, physiological, and biochemical criteria. The pure cultures were put onto NA plates, Nutrient Broth (NB), NA slants, and incubated at 37 °C to determine the colony morphology, and growth pattern of each isolate. Different biochemical tests were performed, i.e., Gram staining and microscopic analysis using a binocular microscope (Model: EC.1152), motility test, catalase test, oxidase test, urease test, indole production, methyl red, Voges-Proskauer, citrate (IMViC) test, gelatin hydrolysis test, and fermentation of carbohydrates such as glucose, sucrose, maltose, and mannitol. Gram staining and motility test were conducted for studying the physiological characteristics of the isolates. The characteristics were evaluated and compared to the standard description according to the Microbiology: A Laboratory Manual [45].

### 2.5 Evaluation of Cr tolerance

The maximum tolerance concentration (MTC) of Cr was determined following the streak plate method as previously described [46] with modification. Cr-tolerant isolates were grown on Cr-incorporated media by gradually increasing its concentration on NA plates. Bacteria inoculated plates were incubated for 10 days at 37 °C. The MTC was defined as the highest concentration of metal which supports the growth in the medium [47]. Basic chromium sulfate and potassium dichromate were used as a source of Cr(III) and Cr(VI) in the medium with the starting concentration of 100 ppm for each. Both positive and negative controls were also employed. A metal-deficient medium that had been inoculated with the bacterium served as a positive control. An absence of the bacterium in a metal-supplemented medium was the negative control.

### 2.6 Molecular and bioinformatic identification of gelatin hydrolyzing bacteria

The isolated bacteria were cultured in the NB medium and incubated overnight at 37 °C. From each bacterial isolate, genomic DNA was extracted following the boiling procedure outlined by Queipo-Ortuño, Maria Isabel et al., with some modifications [48]. In a centrifuge tube, 1 mL of broth culture was taken and centrifuged for 5 min at 10,000 rpm. After removing the supernatant, the pellet was re-suspended in water suitable for molecular biology. This re-suspended pellet was then heated for 10 min at 100 °C, cooled on ice for 10 min, then centrifuged for 10 min at 10,000 rpm. Finally, a supernatant containing genomic DNA from bacteria was collected and stored at a temperature of −20 °C [48]. Universal primers 27F (5′-AGAGTTTGATCCTGGCTCAG-3′) and 1492R (5′-TACGGTTACCTTGTTACGACTT-3′) were used to amplify the 16S rRNA gene [49]. In a final volume of 40 µL, PCR reactions were carried out. This volume included 20 µL of PCR master mix (Promega, USA), 4 µL of DNA, 0.5 µL of each primer 27F and 1492R, and 15 µL of nuclease-free water. The PCR was carried out using the thermocycling parameters described by Plestenjak et al. [50] with some modifications: the denaturation process began with 5 min at 95 °C, followed by 35 cycles of 45-second denaturation at 95 °C, 1 min of annealing at 55 °C, and 2 min of extension at 72 °C. The final extension step was 10 min at 72 °C to finish the cycling. In order to confirm the presence of around 1500 bp PCR products, the products were run through a 1.5 % agarose gel electrophoresis, stained with ethidium bromide, and visualized by a gel documentation system (AZURE biosystems, model-c200) using 1 kb DNA ladder (Promega, USA) **[36]**. Upon the confirmation of their presence, the amplified PCR products were sequenced, and the chromatogram sequencing files were edited using a chromatogram viewer, including FinchTV 1.4.0. and BioEdit. Using the National Centre for Biotechnology Information's (NCBI) Basic Local Alignment Search Tool (BLAST) facility, the obtained sequences of 16S rRNA genes were compared with the 16S rRNA gene sequences of other organisms that had previously been submitted to the GenBank database in order to identify bacterial species [51] (https://www.ncbi.nih.gov/BLAST/). The phylogenetic tree has been generated to determine the taxonomic relationships between the sequence of 16S rRNA genes of the potential isolates with reference sequences in GenBank using MEGA-X software, version 10.1.8. After aligning the sequences using the ClustalW method, the neighbor-joining algorithm and the Jukes-Cantor distance estimate method were used to build the tree, with bootstrap analyses conducted for 100 replicates [52]. The gene sequences of gelatin hydrolyzing bacterial isolates were submitted to NCBI and GenBank (USA) and Accession Numbers were obtained for the promising bacterial isolates.

### 2.7 CTLW biodegradation application

The three potential isolates were applied for the biodegradation of CTLW as previously described [4] with modifications. 10 mL sterile NB was inoculated with a single colony of BI 4, 5, 7, and incubated at 37 °C for 24 h. From that broth culture 1 % v/v 30 mL overnight grown culture was prepared for each bacterial isolate. 0.3 g of dried CTLW (1 % w/v) were measured for each culture followed by individual wrapping in foil envelopes and autoclaved at 121 °C for 15 min to prevent unwanted contamination. Autoclaved CTLW was taken in 1 % v/v inoculum culture and biodegradation experiment was carried out for 7 days at 37 °C. CTLW in NB without bacterial inoculum was served as control experiment. After 7 days, the liquefied media were filtered using Whatman grade 1 qualitative filter paper 150 mm diameter. Filter papers containing the non-degraded CTLW were dried, and the biodegradation percentage was assessed for each isolate. Each experiment was repeated three times in this study. The percentage of biodegradation of the CTLW was calculated using the following formula:

% Degradation =.(Wi-Wd)/Wi×100

where Wi is the initial weight of CTLW and Wd is the dry weight of non-degraded CTLW.

### 2.8 Statistical analysis

The data were analyzed by using MS Excel 2016 and presented as mean ± standard error (SE) of three replicates. Statistical analyses were performed using analysis of variance (ANOVA) followed by the Tukey HSD multiple comparison test to detect the significant differences (P < 0.05) between means by using SPSS vs 25.0.

## 3 Results

### 3.1 Physico-chemical analysis of DW, ECW and CTLW

The findings of the physico-chemical analysis are presented in Table 1 for DW, ECW, and CTLW. Temperature and pH were measured during sample collection. TDS and total Cr content were measured in the laboratory. In this study, the temperature of the liquid samples was around 21 °C while the range of pH values was 7.52 to 7.93. DW contained the highest TDS concentration, accounting for 11,800 ppm. On the contrary, the lowest TDS was recorded for ECW 1, and it was only 37 ppm. Temperature, pH, and TDS were not determined for CTLW. In the case of total Cr concentration, CTLW contained the highest amount, which accounted for 17,765 ppm. Among the liquid samples, DW had the largest concentration, which was precisely 2 ppm. The Cr concentrations in ECW 1, ECW 2, and ECW 3 were almost similar.

**Table 1:** Physico-chemical parameters of samples.

Sample	Physical parameter			Metal concentration (ppm)
	Temp. (°C)	pH	TDS (ppm)	Total Cr
DW	21.3	7.52	11,800	2.0
ECW 1	21.3	7.76	37	0.30
ECW 2	21.2	7.78	118	0.40
ECW 3	21.3	7.93	574	0.30
CTLW	ND	ND	ND	17,765.0

ND= Not Determined.

### 3.2 Isolation of Cr(III) tolerant bacteria

Visual observation of growth in Cr supplemented (100 ppm) NA medium after 2–3 days of incubation indicated that the collected samples contain chromium-tolerant bacteria. After primary screening, eight Cr(III) tolerant bacteria were isolated from tannery wastes in the present study. Among them, three were isolated from CTLW, two were from DW, and three bacteria were isolated from ECW. The bacterial isolates (BI) were arbitrarily named BI 1, BI 2, BI 3, BI 4, BI 5, BI 6, BI 7, and BI 8.

### 3.3 Morphological and biochemical characterization

Bacterial isolates were characterized by morphological, cultural, physiological, and biochemical properties. The colony morphology and growth pattern of each isolate were observed and recorded. Bacterial isolates showed different morphological properties and growth patterns in nutrient agar plate and nutrient agar slant, respectively. However, all of the isolates produced sediment when cultured in nutrient broth (Table 2).

**Table 2:** The growth pattern and colony morphology of isolates in different media.

Bacterial isolates	Colony morphology					Growth pattern in	
	Color	Shape	Elevation	Margin	Surface	Nutrient broth	Nutrient agar slant
BI 1	Creamy white	Irregular	Convex	Entire	Smooth	Sediment	Echinulate
BI 2	Creamy white	Round	Convex	Entire	Smooth	Sediment	Filiform
BI 3	Pink	Irregular	Raised	Undulate	Wrinkle	Sediment	Beaded
BI 4	Milk white	Irregular	Umbonate	Lobate	Wrinkle	Sediment	Arborescent
BI 5	White	Irregular	Flat	Lobate	Wrinkle	Sediment	Filiform
BI 6	Creamy yellow	Irregular	Raised	Lobate	Wrinkle	Sediment	Arborescent
BI 7	Milk white	Irregular	Umbonate	Lobate	Wrinkle	Sediment	Arborescent
BI 8	Pink	Irregular	Raised	Undulate	Wrinkle	Sediment	Filiform

**Fig. 1:**
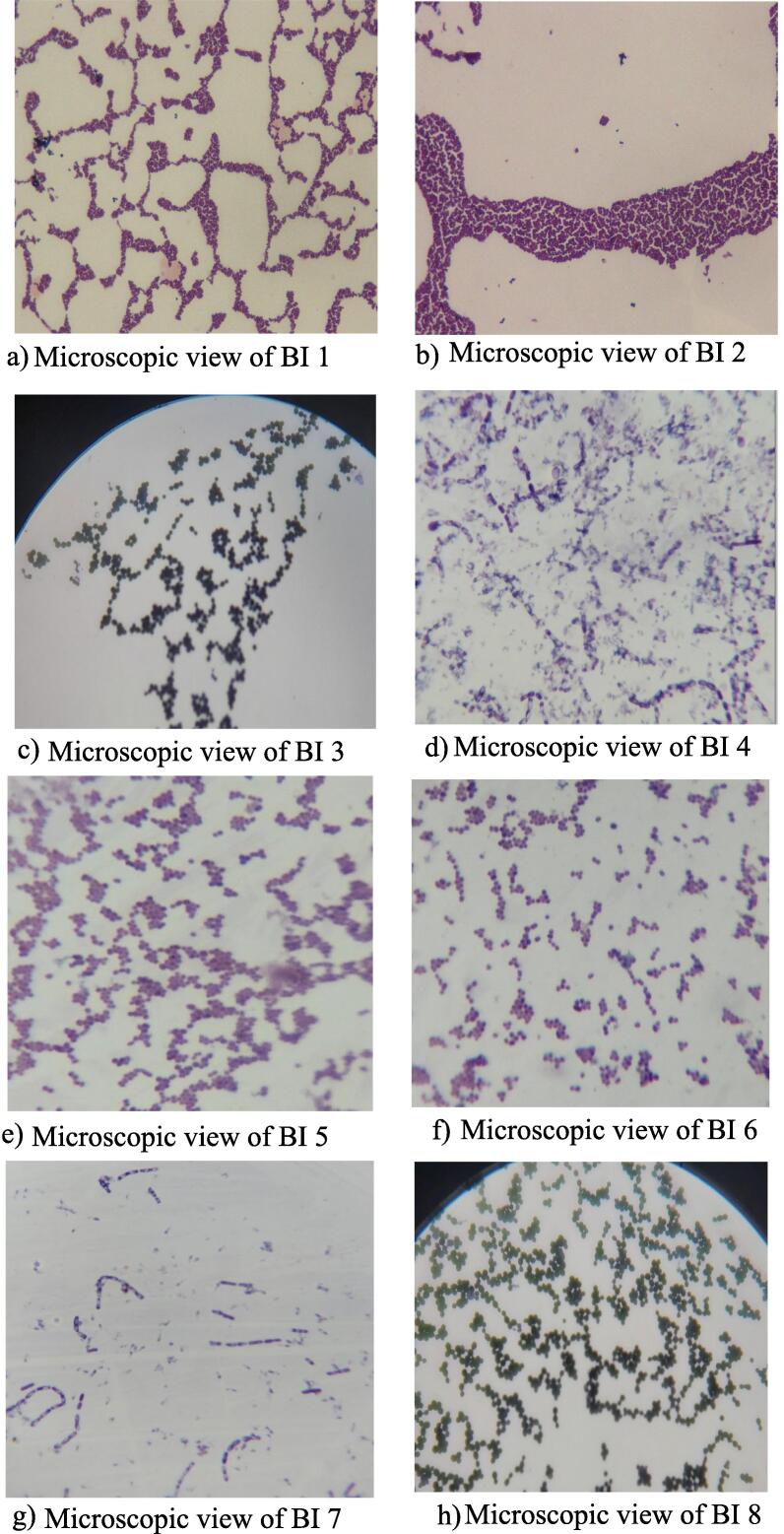
Microscopic view of bacterial isolates (BI) a-h.

The isolates were classified as Gram-positive or Gram-negative bacteria according to the kind of cell walls, their shape, and the arrangement of their cells, as shown by Gram staining. All of the bacterial isolates were found Gram-positive. The cells of BI 1, 2, 3, 5, 6, and 8 (Fig. 1; a, b, c, e, f, and h respectively, and Table 3) were found as cocci with irregular clusters while BI 4 and 7 were rod-shaped and their cells were arranged singly or in short chain (Fig. 1; d and g respectively, and Table 3). Bacterial isolates 1, 3, 4, 5, 6, 7, and 8 were motile while BI 2 was found as non-motile (Table 3).

**Table 3:** Morphological, physiological, staining, and biochemical characteristics of isolates.

Features/tests	Bacterial isolates (BI)							
Morphological/ Physiological/ staining/biochemical characteristics	BI 1	BI 2	BI 3	BI 4	BI 5	BI 6	BI 7	BI 8
Cell shape	Cocci	Cocci	Cocci	Rod	Cocci	Cocci	Rod	Cocci
Cell arrangement	Irregular clusters	Irregular clusters	Irregular clusters, short chains, and pairs	Chain and single	Irregular clusters, and short chain	Irregular clusters, and short chain	Chain and single	Irregular clusters, short chains, and pairs
Motility	(+)	(−)	(+)	(+)	(+)	(+)	(+)	(+)
Gram staining	(+)	(+)	(+)	(+)	(+)	(+)	(+)	(+)
Catalase test	(+)	(±)	(+)	(+)	(±)	(+)	(+)	(+)
Oxidase test	(−)	(−)	(−)	(+)	(−)	(−)	(+)	(−)
Urease test	(−)	(−)	(+)	(+)	(−)	(−)	(−)	(+)
Indole test	(−)	(−)	(−)	(−)	(−)	(−)	(−)	(−)
Methyl red test	(−)	(−)	(−)	(+)	(−)	(−)	(+)	(−)
Voges-Proskauer test	(−)	(−)	(−)	(+)	(−)	(−)	(+)	(−)
Citrate test	(−)	(−)	(−)	(−)	(−)	(+)	(−)	(−)
Gelatin hydrolysis test	(−)	(−)	(−)	(+)	(±)	(−)	(+)	(−)
Glucose fermentation with gas production	(−)(−)	(−)(−)	(−)(−)	(+)(+)	(+)(+)	(+)(−)	(+)(+)	(+)(±)
Mannitol fermentation with gas production	(−)(−)	(−)(−)	(−)(−)	(±) (−)	(+)(−)	(±)(−)	(±)(−)	(−)(−)
Sucrose fermentation with gas production	(+)(−)	(−)(−)	(−)(−)	(+)(−)	(+)(±)	(+)(−)	(+)(−)	(−)(−)
Maltose fermentation with gas production	(−)(−)	(−)(−)	(−)(−)	(+)(−)	(+)(−)	(+)(−)	(+)(−)	(+)(±)

+,positive; ±,weakly positive; −,negative.

Biochemical characteristics of BI 1, 2, 3, and 8 showed a similar response except the ability to utilize urea, and the fermentation of glucose, sucrose and maltose. Glucose and maltose fermentation were reported for BI 8 with weak gas production ability. Sucrose fermentation was observed for BI 1 without gas production. Urease activity was shown by BI 3 and BI 8, as they were able to utilize urea as sole source of nitrogen. None of the tested carbohydrate fermentation was observed for BI 2 and BI 3 (Table 3). Identical results for biochemical tests such as catalase, oxidase, methyl red, Voges-Proskauer, and gelatin hydrolysis were observed for BI 4 and BI 7. Indole and citrate test results were negative for both of them. For the urease test, BI 4 utilized urea, while BI 7 did not. Glucose fermentation was reported for BI 4 and BI 7 with gas production, while they responded weakly to ferment mannitol without gas production. In addition, both BI 4 and BI 7 were reported to ferment sucrose and maltose without gas production (Table 3).

Other than citrate and gelatin hydrolysis test, BI 5 and BI 6 were also found to have similar biochemical characteristics. Citrate utilization was reported by BI 6, but BI 5 could not do that. Gelatin hydrolysis activity was not shown by BI 6, while BI 5 showed weak gelatin hydrolysis ability (Table 3). Their carbohydrate fermentation pattern was also in a similar line. Both were able to ferment all of the tested carbohydrates with or without gas production. Comparing the results of morphological, physiological, staining, and biochemical characteristics with the standard description of the seventh edition of Bergey's Manual of Determinative Bacteriology and also with the reports of previous investigations, the isolates were tentatively identified up to genus level as *Micrococcus* sp. (BI 1, 2, 3 and 8); *Bacillus* sp. (BI 4 and 7); *Enterococcus* sp*.* (BI 5 and 6).

### 3.4 The isolates' maximum tolerance concentration (MTC) for Cr(III) and Cr(VI)

**Fig. 2:**
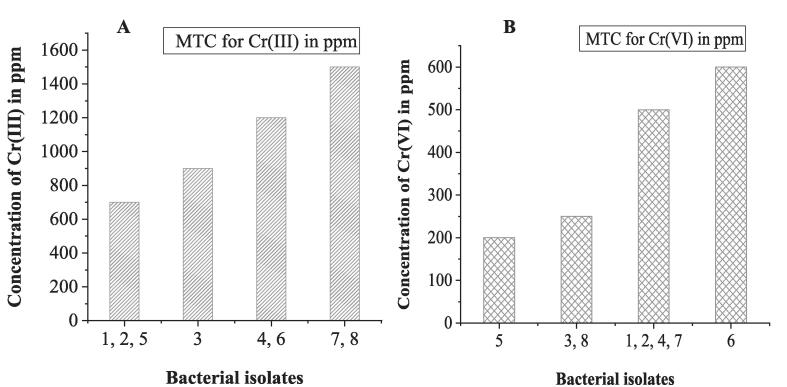
MTC of bacterial isolates to Cr(III) [A] and Cr VI) [B].

The isolates' tolerance to Cr(III) and Cr(VI) was evaluated using NA medium amended with varying doses of basic chromium sulfate and potassium dichromate as a source of Cr(III) and Cr(VI), correspondingly. The MTC of Cr(III) for BI 1, 2, and 5 was 700 ppm [Fig. 2 (A)], and for BI 3, it was 900 ppm [Fig. 2 (A)]. BI 4 and 6 were reported to grow at the maximum tolerance concentration of 1200 ppm Cr(III) [Fig. 2 (A)]. The MTC of Cr(III) for BI 7 and 8 was the highest, which was 1500 ppm [Fig. 2 (A)]. On the other hand, for Cr(VI), BI 1, 2, 4, and 7 tolerated up to 500 ppm [Fig. 2 (B)]. BI 6 was found to have the MTC of Cr(VI) at 600 Cr [Fig. 2 (B)]. The MTC of Cr(VI) for BI 3, 8, and BI 5 was 250 ppm and 200 ppm, respectively [Fig. 2 (B)].

### 3.5 Molecular and bioinformatic characterization

**Fig. 3:**
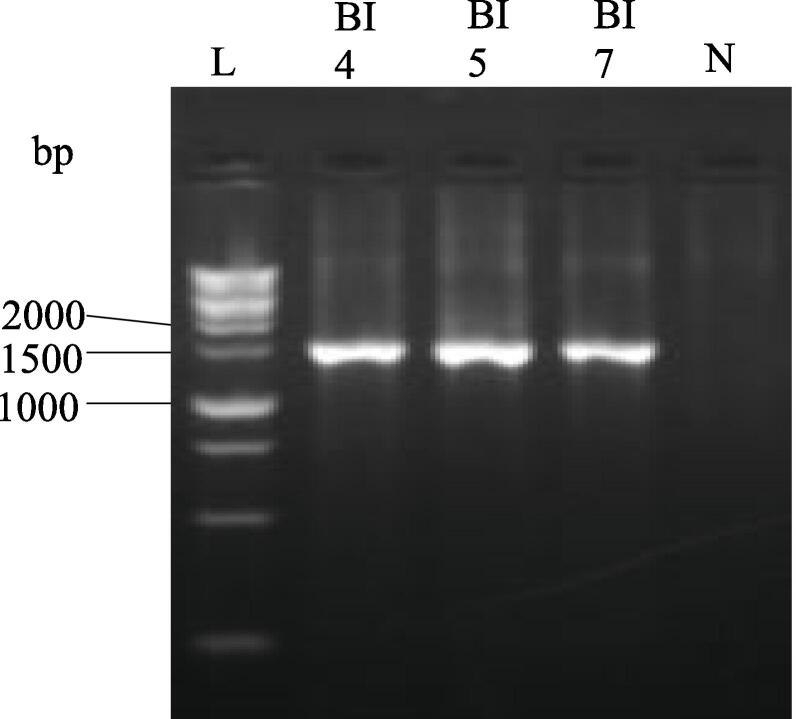
PCR amplification of 16S rRNA gene. Lanes: L (1 kb ladder), BI 4, BI 5, BI 7 (Bacterial isolates 4, 5, 7 respectively), and N (negative control).

Genomic DNA was extracted from the pure culture, and PCR was performed. Visualization of PCR amplified product by a gel documentation system exhibited the 16S rRNA gene amplification by BI 4, BI 5, and BI 7 at about 1500 base pair (bp) when compared with 1 kb DNA ladder (Fig. 3).

**Fig. 4:**
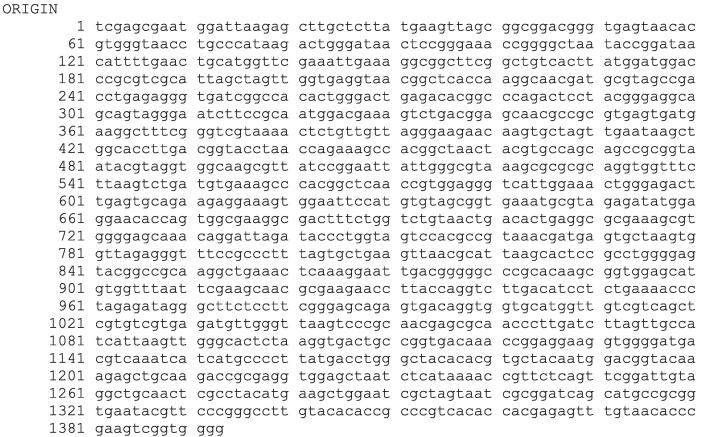
16S rRNA sequence of bacterial isolate (BI) 4.

**Fig. 5:**
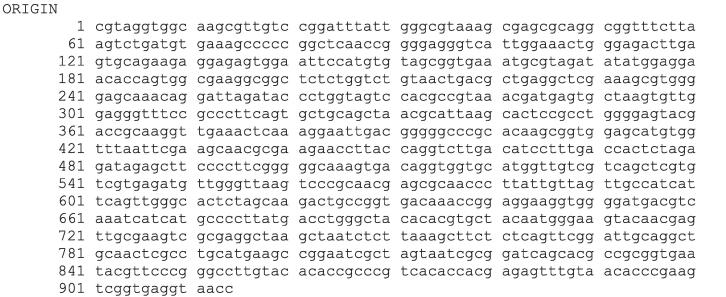
16S rRNA sequence of bacterial isolate (BI) 5.

**Fig. 6:**
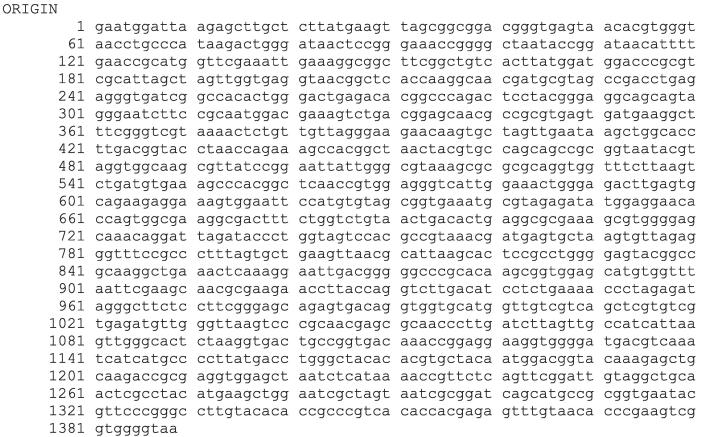
16S rRNA sequence of bacterial isolate (BI) 7.

Using the forward and reverse primer, the PCR product was bi-directionally sequenced. The sequences of BI 4, BI 5 and BI 7 have been presented in Fig. 4, Fig. 5, Fig. 6, correspondingly.

**Fig. 7:**
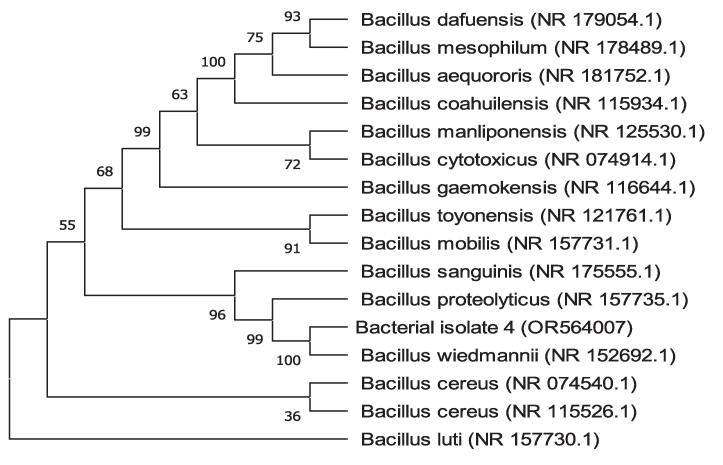
The phylogenetic tree of *Bacillus wiedmannii* (OR564007) on the basis of patterns and genetic relationships. Accession numbers for GenBank are indicated by numbers in brackets.

**Fig. 8:**
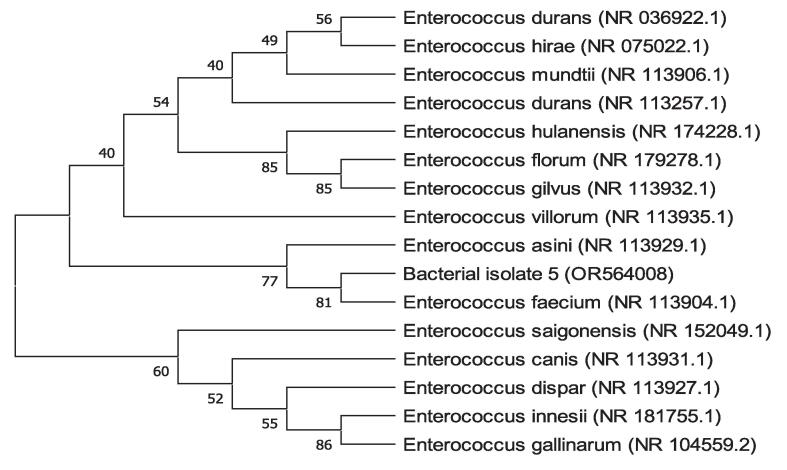
The phylogenetic tree of *Enterococcus faecium* (OR564008) on the basis of patterns and genetic relationships. Accession numbers for GenBank are indicated by numbers in brackets.

**Fig. 9:**
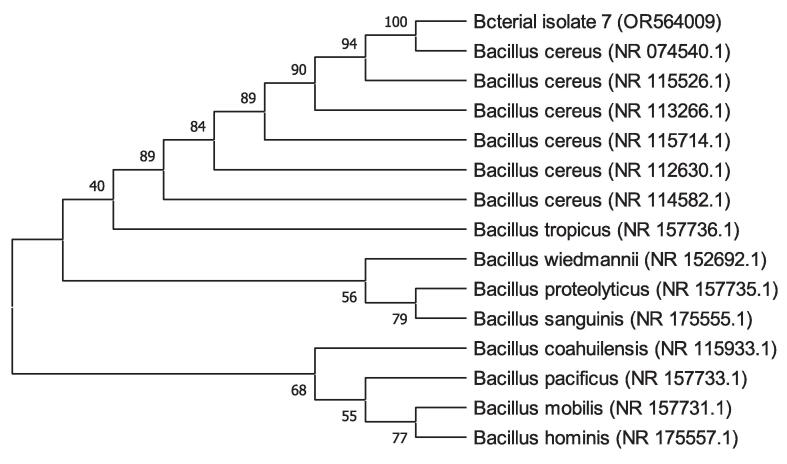
The phylogenetic tree of *Bacillus cereus* (OR564009) on the basis of patterns and genetic relationships. Accession numbers for GenBank are indicated by numbers in brackets.

Following software analysis (FinchTV 1.4.0 and BioEdit) of the 16S rRNA sequences, the BlastN tool was used to analyze the similarity and homology with the preexisting sequences stored in the National Center for Biotechnology Information (NCBI) database. The alignment findings indicate that the sequences of 16S rRNA genes of bacterial isolates 4, 5, and 7 exhibited a significant degree of similarity to *Bacillus wiedmannii* (NR_152692.1), *Enterococcus faecium* (NR_113904.1), and *Bacillus cereus* (NR_074540.1), respectively. The alignment revealed a query coverage of 100 % for all of the bacterial isolates while a percent identity of 99.93 % for BI 4 and 100 % for both BI 5 and 7, respectively. The phylogenetic tree analysis revealed that bacterial isolates 4, 5, and 7 shared a cluster with *Bacillus wiedmannii*, *Enterococcus faecium*, and *Bacillus cereus,* as depicted in Figs. 7, 8, and 9, respectively. Therefore, the BI 4, 5, and 7 were identified as *Bacillus wiedmannii* (OR564007), *Enterococcus faecium* (OR564008), and *Bacillus cereus* (OR564009), respectively. Accession numbers for GenBank are indicated by numbers in brackets, respectively.

### 3.6 CTLW biodegradation

The biodegradation percentage was assessed based on dry weight matter by measuring the loss of weight of the CTLW. *Enterococcus faecium* degraded the highest amount of CTLW, which was about 0.296 ± 0.0009 g. The lowest degradation was recorded for *Bacillus wiedmannii*, and it was accounted for 0.294 ± 0.0006 g. However, there was not so much variation among the degradation amount of CTLW (Table 4). The order of CTLW biodegradation percentage was 98.67 %>98.33 %>98.00 % for *Enterococcus faecium > Bacillus cereus > Bacillus wiedmannii*, correspondingly.

**Table 4:** Degraded amount of CTLW by bacterial isolates with gelatin hydrolysis ability.

Bacterial isolate (BI)	Degraded amount of CTLW in g
4 (*Bacillus wiedmannii*)	0.294 ± 0.0006^a^
5 (*Enterococcus faecium*)	0.296 ± 0.0009^a^
7 (*Bacillus cereus*)	0.295 ± 0.0008^a^

Values are means of triplicate determination (n = 3) ± standard error. Mean values in columns with superscript letters (a) indicate that they fall in the same group. Mean values are not significantly different (p > 0.05).

## 4 Discussion

In this study, three types of samples were collected, and the TDS concentration ranged from 37 to 11,800 ppm (Table 1), with one sample exceeding the standard permissible limits (2100 ppm) for discharging into inland surface water established by the Inland Surface Water-Bangladesh Standards- Environmental Conservation Rules, 1997 (ISW-BDS-ECR 1997) [53]. A previous study also found TDS concentration higher than the standard permissible limit in the tannery wastewater [54]. The pH values ranged between 7.52 and 7.93 (Table 1). All of the analyzed liquid samples were within the range of acceptable standards (pH 6–9) established by ISW-BDS-ECR (1997) [53]. When analyzing the tannery effluent from Hazaribgh, Bangladesh, Rouf et al., also observed an experimental average pH value of 7.16 [54]. In the presented study, higher pH and TDS were reported for the sample DW, and previous studies have also reported similar findings in tannery effluent 55, 56.

The temperature of water was similar regardless of sampling locations. In the case of total chrome concentration, ECW 1, ECW 2, and ECW 3 contained Cr nearly the permissible limit (0.50 ppm) for discharging into inland surface water set by ISW-BDS-ECR (1997) [53]**,** which was 0.30, 0.40 and 0.30 ppm, respectively (Table 1). Among the liquid samples, only the DW sample contained a Cr concentration of 2.0 ppm, which was about four times the allowable upper maximum. Cr concentration was the highest in the CTLW sample, and the amount was 17,765 ppm. Ahmed et al., also reported that the wet blue shaving dust (WSD) contained 29,854.4 ppm of Cr [57].

The optimal method for choosing metal-resistant strains of bacteria for bioremediation and the removal of heavy metals is to isolate them from tannery environments [58]. In the present study, eight bacteria resistant to Cr(III) were isolated from different tannery waste samples. All isolates were successfully grown in culture media amended with Cr. Several studies have demonstrated that bacteria capable of thriving at high Cr(VI) concentrations and reducing to Cr(III) can be isolated from a range of Cr-containing wastewater [50]. However, a few have been reported on Cr(III) tolerant bacterial isolation from tannery wastes [47]. Following isolation, morphological and biochemical tests, most notably the gelatin hydrolysis test, were conducted to characterize the bacterial isolates. Two morphological variations were observed irrespective of bacterial isolates. BI 1, 2, 3, 5, 6, and 8 were found as coccus, while BI 4 and 7 had different morphological characteristics, and they were observed as rod-shaped. Based on gram staining, the bacterial isolates were all gram-positive.

In the laboratory, catalase test, oxidase test, urease test, indole production, methyl red, Voges-Proskauer, citrate (IMViC) test, gelatin hydrolysis test, and fermentation of carbohydrates such as glucose, sucrose, maltose, and mannitol were conducted for biochemical analysis of those isolates. In some previous investigations, similar tests were also performed to determine the biochemical characteristics of bacteria 59, 60, 61, 62, 63, 64. In this study, isolated bacteria were identified down to the genus level based on the results of the gelatin hydrolyzing activity, and other biochemical and physiological analyses. BI 1, 2, 3, and 8 were identified as *Micrococcus* sp.*,* BI 4 and 7 as *Bacillus* sp.*,* and BI 5 and 6 as *Enterococcus* sp. Among the isolated bacteria, BI 4, 5, and 7 were able to hydrolyze gelatin, which means they were able to produce gelatinase and utilize gelatin as a substrate. Previous studies reported that hydrothermal treatment, such as autoclaving, can result in the formation of gelatin from chrome-tanned leather waste 18, 65. Greenwell et al., showed that gelatin acts as a nutritious component, and the leather's gelatin content could have accelerated the growth of bacteria [4]. This facilitated the production of enzymes such as protease, and the degradation of leather waste 4, 18. Those studies support that the bacteria having the ability to utilize gelatin can also be efficient in using hydrothermally treated Cr-tanned leather waste as a source of nutrients, and stimulate its degradation. In order to the microbial growth of chrome-tanned leather waste and its degradation, the strain must have the ability to grow in an environment that contains high concentrations of Cr 18, 20. Therefore, knowing the maximum tolerance concentration (MTC) of bacterial isolates against Cr is an urgent need. To meet that necessity, the growth of bacterial isolates was tested in response to varied Cr concentrations, including both Cr(III) and Cr(VI). In this study, distinct MTC levels were observed among the isolated species. For Cr(III), BI 7 and 8 showed strong resistance capability at 1500 ppm, while the MTC of BI 4 and 6 were found at 1200 ppm. BI 3 exhibited an MTC of 900 ppm, and for BI 1, 2, and 5, the MTC was 700 ppm. Sundar et al., also conducted an investigation to determine the MTC levels of bacterial isolates against Cr(III), and some isolates were found to have tolerances between 500 and 1500 ppm [47].

On the other hand, for Cr(VI) MTC determination, BI 6 exhibited growth at the highest concentration of Cr(VI), which was 600 ppm. Bacterial isolates 1, 2, 4, and 7 were able to grow at the concentration of Cr(VI) 500 Cr. The MTC for BI 3 and 8 was 250 ppm, while BI 5 was 200 ppm. A previous study reported that bacteria showed a higher tolerance ability to Cr(III) than Cr(VI) [18].

Phenotypic and conventional biochemical descriptions are sometimes unable to differentiate bacteria much more precisely among species. However, the 16S rRNA gene sequences make it possible for bacteria to distinguish between species and subspecies levels [66]. Therefore, accurate identification of bacteria through the 16S rRNA gene sequencing is highly important for their deposit in a database such as the NCBI GeneBank. In this study, three potential bacterial isolates with gelatin hydrolyzing activity were considered for 16S rRNA gene sequencing. Accession numbers were obtained following the submission of the sequences of all three bacterial isolates to NCBI, GenBank (USA). Based on the 16S rRNA gene sequence and phylogenetic analysis, those three bacterial isolates, namely BI 4, 5, and 7 have been identified as *Bacillus wiedmannii* (Accession No: OR564007; Figure: 7), *Enterococcus faecium* (Accession No: OR564008; Figure: 8), and *Bacillus cereus* (Accession No: OR564009; Figure: 9), respectively. The 16S rRNA gene sequence analysis, as compared to traditional approaches, has been shown to be preferable for identifying bacteria in a number of earlier investigations 35, 67, 68. In addition, phylogenetic analysis and the sequencing of the 16S rRNA gene were regarded as powerful tools for identifying bacterial isolates [52].

In this study, the bacterial isolates with gelatin hydrolysis ability were applied for CTLW biodegradation experiments. The biodegradation results showed that the mean value of biodegraded amount of CTLW was not significantly different (p > 0.05) for all the three tested bacteria (Table 4). This indicates that *Enterococcus faecium, Bacillus cereus,* and *Bacillus wiedmannii* have similar biodegradation ability with the biodegradation percentages of 98.67 %, 98.33 % and 98.00 %, respectively. Therefore, this study signifies the hypothesis that bacteria with gelatin hydrolyzing activity can be the potential to biodegrade hydrothermally treated CTLW. A previous research showed that bacterium with gelatinase activity was able to degrade autoclaved leather dust 4, 69. The species described in this study were more potential in degrading hydrothermally treated CTLW than *A. carbonarius*, which took 12 days to degrade 1 % Cr shavings [19]. Another previous study reported that *Bacillus subtilis* P13 degraded 1 % chrome shavings in 24 h, however, the degradation percentage was only 90 % [18].

## 5 Conclusion

CTLW is a hazardous material which causes environmental pollution, and affects human health. Conventional methods of CTLW management also cause secondary pollution. An effective approach of management can be the utilization of bacteria, therefore, isolation and molecular characterization of potential bacteria is an urgent need. Eight Cr-tolerant bacteria were isolated from the collected tannery waste samples. Among them, three promising bacteria were further screened based on their gelatin hydrolyzing ability. 16S rRNA molecular characterization identified those three isolates as *Bacillus wiedmannii* (Accession No: OR564007), *Enterococcus faecium* (Accession No: OR564008), and *Bacillus cereus* (Accession No: OR564009) for BI 4, 5 and 7, respectively. *Bacillus wiedmannii*, *Enterococcus faecium* and *Bacillus cereus* were able to produce gelatinase as they had been found hydrolyzing gelatin. Therefore, experiments were conducted for establishing their ability to biodegrade hydrothermally treated CTLW. *Enterococcus faecium*, *Bacillus cereus*, and *Bacillus wiedmannii* were almost equally potential for the biodegradation of CTLW. In future experiments, the identified three potential bacteria will be used for the biodegradation of CTLW singly and in combination to establish the optimal biodegradation approach, liberating Cr in the liquefied effluent, consequently, facilitating the recovery of Cr to tackle the waste pollutant and alleviate environmental pollution. This study will help future researchers isolate and identify the Cr-tolerant potential bacterial candidates from tannery waste samples for their feasible applications in waste management.

## CRediT authorship contribution statement

**Shashanka Shekhar Sarker:** Writing – review & editing, Writing – original draft, Visualization, Software, Project administration, Methodology, Investigation, Funding acquisition, Formal analysis, Data curation, Conceptualization. **Md.Murshed Hasan Sarkar:** Writing – review & editing, Software, Data curation. **Shamima Akhter Sharmin:** Writing – review & editing, Investigation. **Nourin Tarannum:** Writing – review & editing, Investigation. **Taslima Akter:** Investigation. **Md.Ashraful Alam:** Investigation, Formal analysis. **Md.Ibrahim Miah:** Writing – review & editing. **Md.Aftab Ali Shaikh:** Writing – review & editing, Visualization, Validation. **Sahana Parveen:** Writing – review & editing, Validation, Supervision, Project administration, Methodology.

## Funding

This work was supported by the Bangladesh Council of Scientific and Industrial Research (BCSIR) as part of the Research and Development (R&D) project. This research received no specific grant from any funding agency in the public, commercial, or not-for-profit sectors.

## Declaration of competing interest

The authors declare that they have no known competing financial interests or personal relationships that could have appeared to influence the work reported in this paper.
